# The adiponectin receptor AdipoR2 and its *Caenorhabditis elegans* homolog PAQR-2 prevent membrane rigidification by exogenous saturated fatty acids

**DOI:** 10.1371/journal.pgen.1007004

**Published:** 2017-09-08

**Authors:** Ranjan Devkota, Emma Svensk, Mario Ruiz, Marcus Ståhlman, Jan Borén, Marc Pilon

**Affiliations:** 1 Department of Chemistry and Molecular Biology, University of Gothenburg, Gothenburg, Sweden; 2 Department of Molecular and Clinical Medicine/Wallenberg Laboratory, Institute of Medicine, University of Gothenburg, Gothenburg, Sweden; University of California San Francisco, UNITED STATES

## Abstract

Dietary fatty acids can be incorporated directly into phospholipids. This poses a specific challenge to cellular membranes since their composition, hence properties, could greatly vary with different diets. That vast variations in diets are tolerated therefore implies the existence of regulatory mechanisms that monitor and regulate membrane compositions. Here we show that the adiponectin receptor AdipoR2, and its *C*. *elegans* homolog PAQR-2, are essential to counter the membrane rigidifying effects of exogenously provided saturated fatty acids. In particular, we use dietary supplements or mutated *E*. *coli* as food, together with direct measurements of membrane fluidity and composition, to show that diets containing a high ratio of saturated to monounsaturated fatty acids cause membrane rigidity and lethality in the *paqr-2* mutant. We also show that mammalian cells in which AdipoR2 has been knocked-down by siRNA are unable to prevent the membrane-rigidifying effects of palmitic acid. We conclude that the PAQR-2 and AdipoR2 proteins share an evolutionarily conserved function that maintains membrane fluidity in the presence of exogenous saturated fatty acids.

## Author summary

Our cells and their internal organelles are bound by membranes composed primarily of phospholipids, i.e. polar molecules containing two fatty acids attached to a hydrophilic head group. The types of fatty acids in phospholipids greatly influence membrane properties: saturated fatty acids make the membranes rigid while unsaturated fatty acids promote fluidity. The fact that dietary fats can be incorporated into cellular membranes poses a serious challenge to the cells: how to regulate membrane composition to compensate for dietary variations? For the present study we used bacteria mutants with different fat compositions as food sources for the nematode *C*. *elegans*, together with assays to determine membrane rigidity and composition, and discovered that the *C*. *elegans* membrane protein PAQR-2 is responsible for detecting membrane rigidification by dietary saturated fatty acids and to promote fatty acid desaturation to restore membrane fluidity. We also studied the human homolog of PAQR-2, a protein called AdipoR2, and showed that it too is essential to prevent membrane rigidification by saturated fatty acids. AdipoR2 and PAQR-2 therefore serve a critical function in cells by acting as evolutionarily conserved regulators of membrane properties.

## Introduction

The adiponectin receptors AdipoR1 and AdipoR2 are members of the PAQR family of proteins characterized by seven transmembrane domains with their N-terminus facing the cytoplasm [1]. PAQR proteins likely act as hydrolases with different specificities [2], and the crystal structure of the AdipoRs suggests that they act as ceramidases [3,4], which is confirmed by enzymatic assays [3] and is conserved in yeast homologs [5,6]. The ceramidase activity, which would produce a signaling sphingosine 1-phosphate as well as a putative fatty acid that may also serve as a signal, has been proposed to mediate the well-established anti-diabetic effects of the AdipoRs [7,8]. Several important questions still remain regarding the mechanisms by which the AdipoRs exert their effects. In particular, we do not know what conditions trigger AdipoR signaling nor the precise cellular consequences of this signaling.

Our previous studies of PAQR-2, an AdipoR homolog in the nematode *C*. *elegans*, showed that it acts as a sensor of plasma membrane rigidity that can restore membrane fluidity by promoting fatty acid desaturation [9–11]. Mutant worms lacking a functional PAQR-2 protein are intolerant of conditions that promote membrane rigidification, such as cold or diets that increase saturated fatty acid (SFA) content in the worms. For example, including small amounts of glucose in the culture plate results in increased SFA content in membrane phospholipids, membrane rigidification and death of the *paqr-2* mutant [11]. The growth and membrane fluidity of wild-type worms is unaffected by glucose. The extreme sensitivity of the *paqr-2* mutant to glucose is particularly interesting given the anti-diabetic properties of the mammalian AdipoRs since it suggests that these proteins may mitigate glucose toxicity specifically by regulating membrane composition [12]. In *C*. *elegans*, the *paqr-2* mutant defects can be suppressed genetically by mutations that result in increased production of unsaturated fatty acids (UFAs) accompanied by normalization of plasma membrane fluidity or, alternatively, by cultivation in the presence of fluidizing amounts of non-ionic detergents [10,11]. The high degree of sequence homology between PAQR-2 and the AdipoRs (53.7% amino acid identity with AdipoR2 over a 283 aa region) suggests that these proteins have the same cellular functions, i.e. act as sensors/regulators of membrane properties. However, this has not yet been demonstrated for the mammalian AdipoRs.

The present study produced two important advances in our understanding of PAQR-2 and AdipoR2. Firstly, we show that PAQR-2 is essential to prevent membrane rigidification by diets containing a high SFA/monounsaturated fatty acid (MUFA) ratio. Secondly, we demonstrate that the mammalian AdipoR2 protein also acts as a regulator of membrane fluidity that counters the rigidifying effects of exogenous SFAs. Regulation of membrane fluidity is therefore an evolutionary conserved function of the PAQR-2/AdipoR proteins that is essential to cellular health in the presence of exogenous SFAs.

## Results

### Glycolysis-related metabolites are toxic to the *paqr-2* mutant

**Fig 1:**
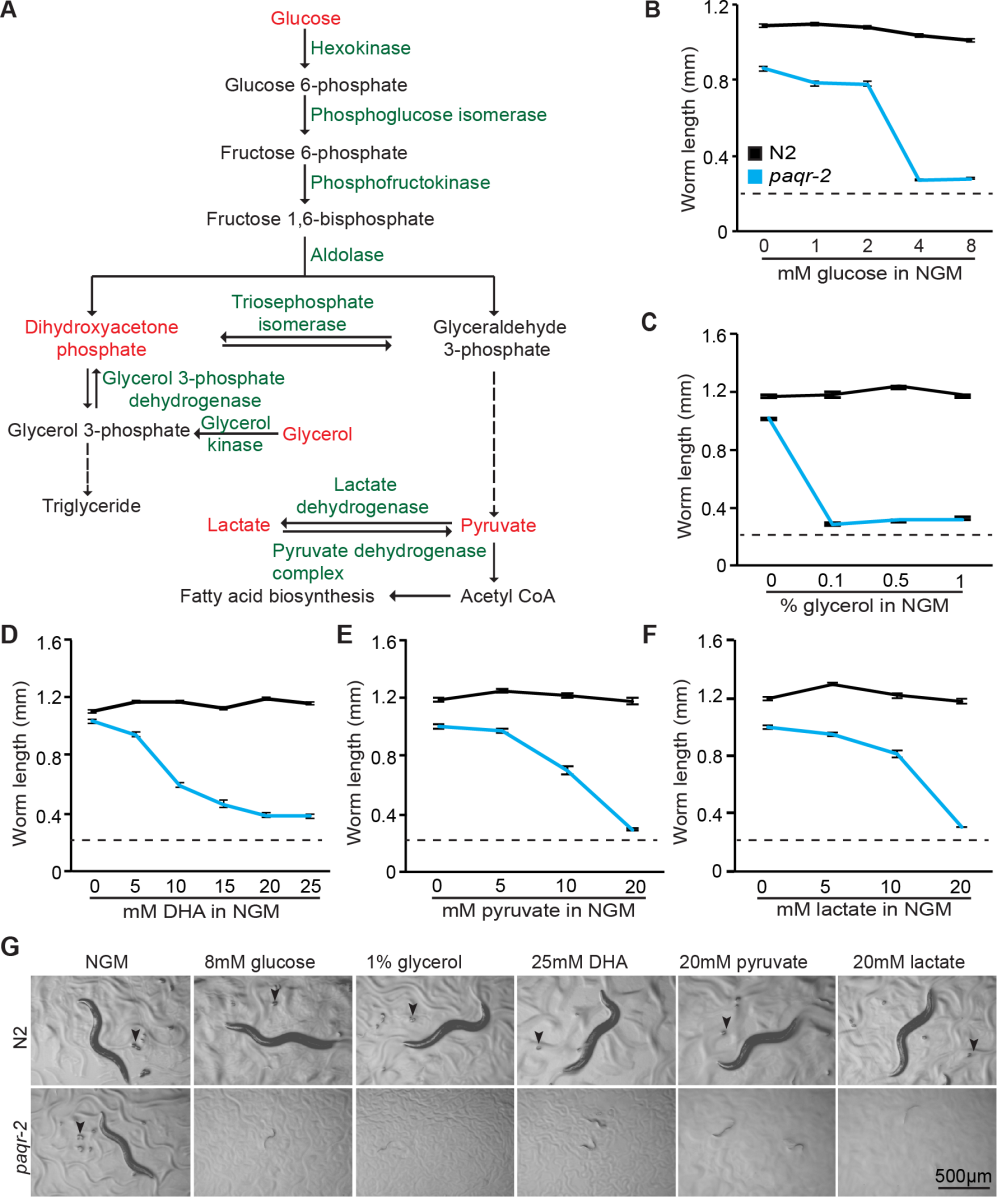
The *paqr-2* mutant is sensitive to several glycolysis-related metabolites. **(A)** Outline of the metabolic pathway connecting the substrates tested, which are indicated with red text. **(B-F)** Length of wild-type N2 and *paqr-2* mutant worms cultivated for 72 hours on various concentrations of metabolite, with representative images shown in **(G).** Note that the initial length of the L1s in these experiments is approximately 0.23 mm (indicated by dashed line in **B-F**): any length greater than this therefore represents growth.

**Fig 2:**
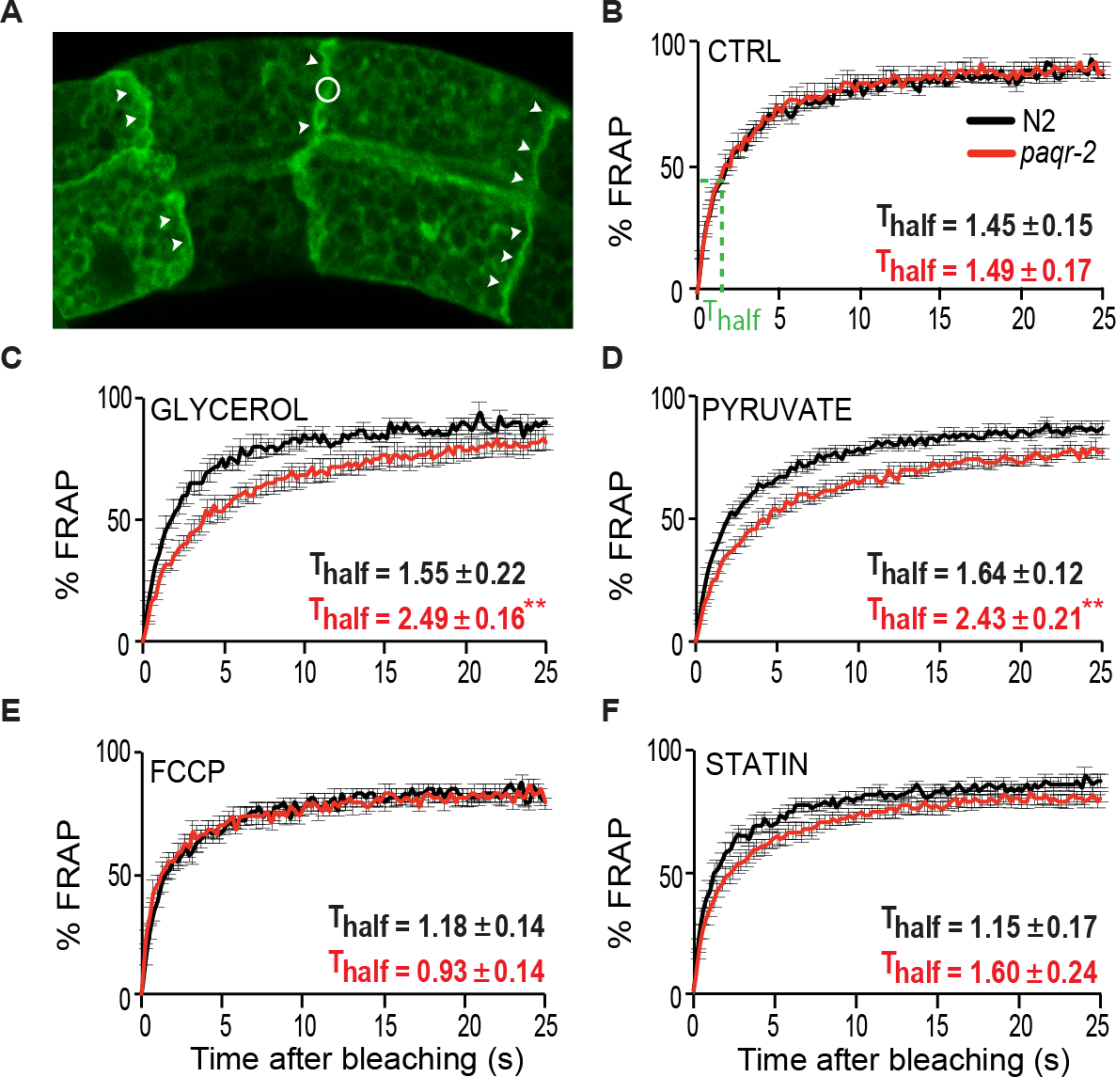
De novo lipogenesis precursors cause reduced membrane fluidity in *paqr-2* mutants as measured by FRAP. **(A)** Confocal image of the prenylated GFP marker enriched on the plasma membrane of intestinal cells. Clear membrane stretches are indicated by arrowheads, and the circle indicate the size of a region bleached during FRAP analysis. **(B-F)** FRAP results from worms grown under different conditions. T_half_ values refer to the time by which half of the maximal fluorescence recovery is achieved (shown in **B**). Note the reduced membrane fluidity of the *paqr-2* mutant grown on glycerol or pyruvate, as evidenced by the lengthened T_half_.

We previously showed that the *C*. *elegans paqr-2* mutant is sensitive to glucose supplementation and that this sensitivity is accompanied by an increase in phospholipid SFAs and membrane rigidity [11]. More recently we discovered that other glycolysis-related metabolites, such as glycerol, dihydroxyacetone, pyruvate and lactate, are also toxic to *paqr-2* (**Fig 1**). This sensitivity is specific to glycolysis-related metabolites: the *paqr-2* mutant is not more sensitive to other types of stressors such as osmotic or oxidative stress (paraquat), inhibition of the respiratory chain or mevalonate pathway, or toxic doses of DMSO (**S1 Fig**). The glucose-related stressors, like glucose itself, also cause rigidification of membranes in the *paqr-2* mutant (**Fig 2**).

### The *E*. *coli* diet is responsible for metabolite toxicity

**Fig 3:**
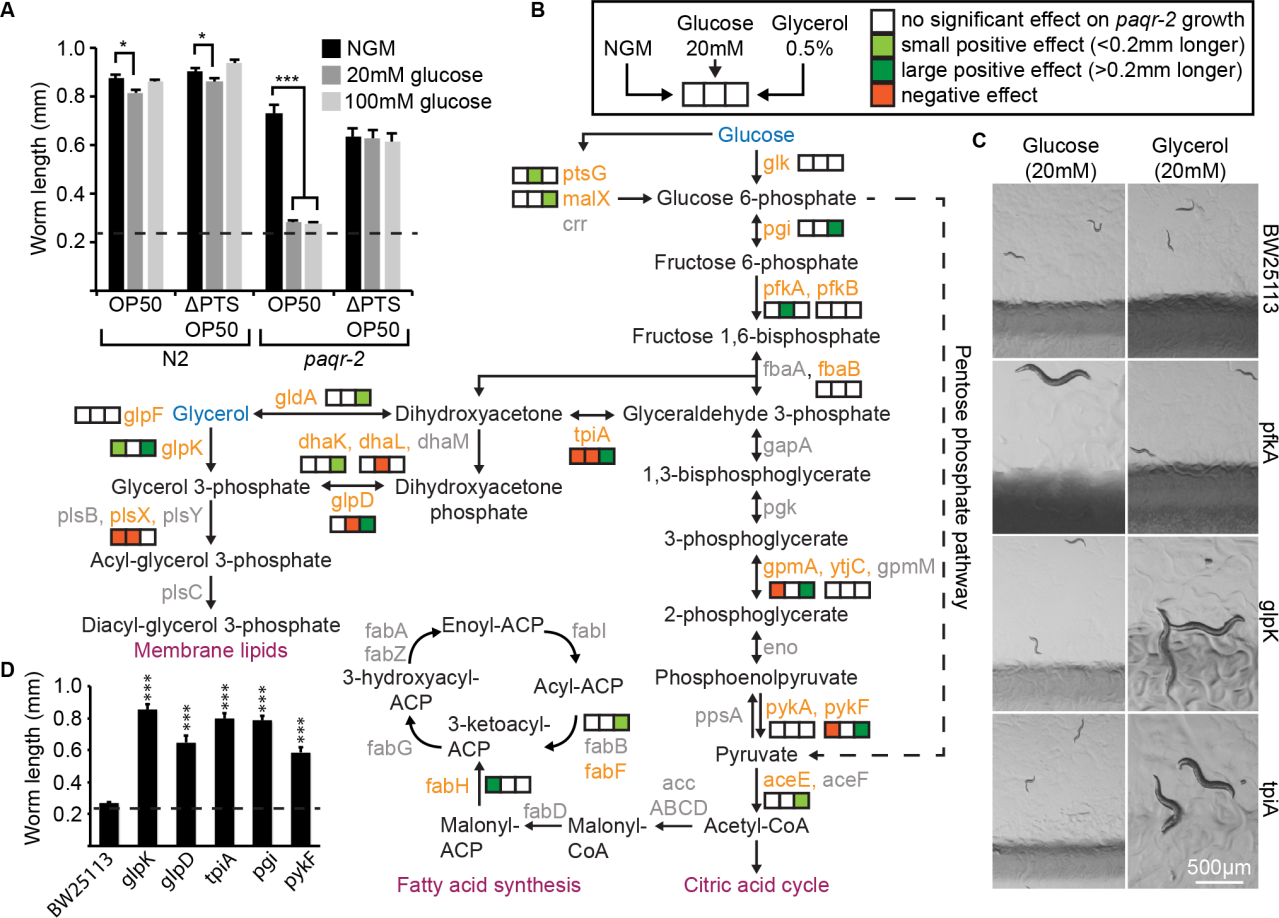
*E*. *coli* is responsible for the conversion of precursors into saturated fatty acids. **(A)** Glucose is not toxic to *paqr-2* mutants fed a glucose uptake-deficient OP50 *E*. *coli* ΔPTS mutant. **(B)** Several mutations that affect the metabolism of fatty acid of precursors abrogate their toxicity. Mutations tested are indicated with orange text. **(C)** Representative images of *E*. *coli* mutants that support growth of the *C*. *elegans paqr-2* mutant in the presence of glucose or glycerol. Note the glossy and discolored appearance of the pfkA mutant. **(D)** Growth of the *paqr-2* mutant on 0.5% glycerol plates seeded with the parental BW25113 and five *E*. *coli* mutants that effectively prevent glycerol toxicity. The dashed line in **(A)** and **(D)** represents the approximate length of the L1s at the start of the experiments.

Others have shown that glucose shortens the lifespan of *C*. *elegans* by acting directly on the worms [13,14]. We were therefore surprised to discover that glucose is not toxic to *paqr-2* mutants grown on an *E*. *coli* strain carrying a ΔPTS mutation that prevents glucose uptake (**Fig 3A)**. This mutation specifically abolishes glucose toxicity, and has no effect on glycerol or pyruvate toxicity (**S2A Fig**). To better understand the role of the bacteria in mediating metabolite toxicity, we took advantage of the Keio collection of *E*. *coli* deletion mutants [15]. This collection is derived from the BW25113 *E*. *coli* strain, a food source that does not attenuate the cold sensitivity or tail tip defects of the *paqr-2* mutant grown on NGM plates (**S2B and S2C Fig**). Of five metabolites tested (glucose, glycerol, dihydroxyacetone, lactate and pyruvate) that are toxic to the *paqr-2* mutant grown on a standard diet of OP50 *E*. *coli*, only two (glucose and glycerol) are toxic to *paqr-2* mutants grown on a diet of BW25113 (**S2D Fig**). Additionally, mutations in several *E*. *coli* genes important for the metabolism of glucose or glycerol abrogated the toxicity of these dietary supplements on *paqr-2* mutants fed BW25113 (**Fig 3B–3D; S3 Fig**). *E*. *coli* metabolism is therefore responsible for the toxicity of several dietary metabolites in the *paqr-2* mutant. Conversely, a normal function of PAQR-2 must be to protect against such dietary toxicity.

### PAQR-2 prevents the lipotoxicity of diets with high SFA/MUFA ratios

**Fig 4:**
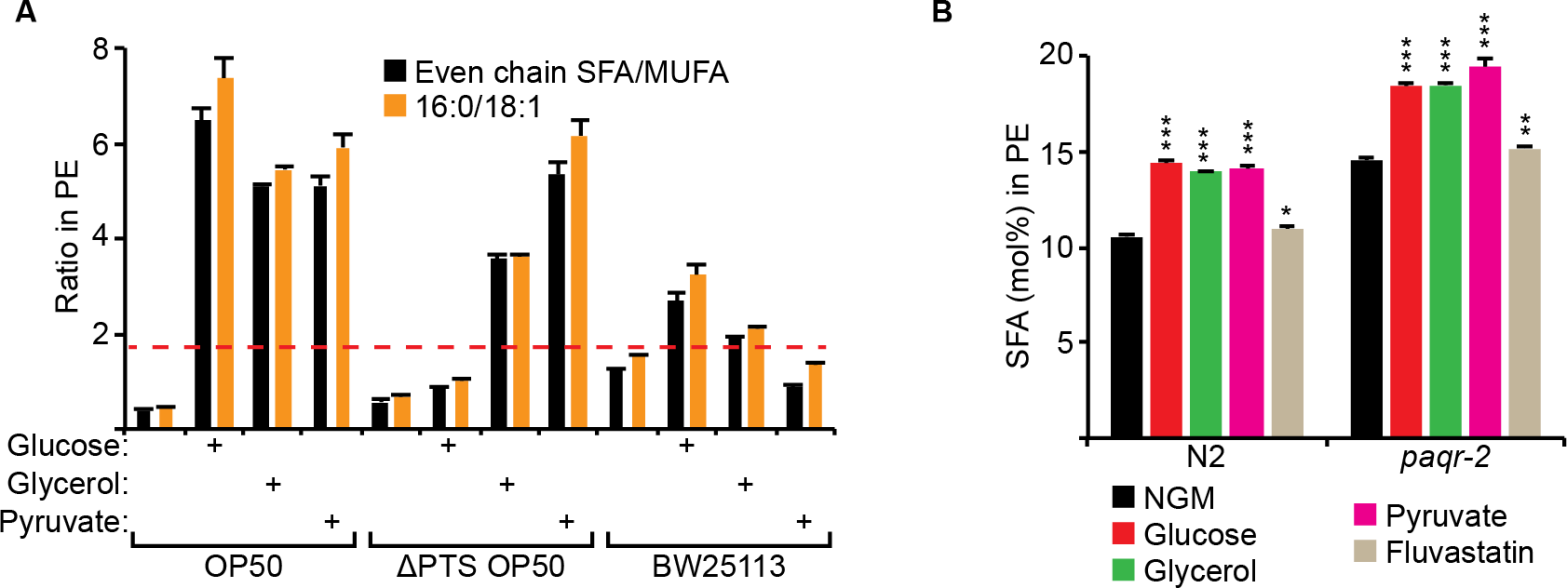
PAQR-2 is required to prevent the toxicity of a diet with a high SFA/MUFA ratio. **(A)** Even chain SFA/MUFA and 16:0/18.1 ratios in the PEs of *E*. *coli* samples from different strains or culture conditions. Conditions where the bars reached higher than the dashed red line (i.e. ratio of ~2:1 or higher) caused growth arrest and lethality of the *paqr-2* mutant. **(B)** SFA fraction within the PEs of worms grown on control plates (NGM) or plates containing the indicated additives. Note the excessive enrichment of SFAs in *paqr-2* mutants cultivated on glucose, glycerol or pyruvate.

The metabolites toxic to *paqr-2* mutants can all be readily converted into acetyl-CoA, a precursor for fatty acid synthesis (**Fig 3B**). To better understand the nature of the dietary toxicity, we analyzed the fatty acid composition of *E*. *coli* grown with different supplements or mutations. We found that dietary *E*. *coli* containing elevated SFA/MUFA ratios are toxic to the *paqr-2* mutant (**Fig 4A**; complete lipidomics values are provided as a separate supplementary dataset file). Specifically, a dietary SFA/MUFA ratio higher than ~1.8 results in an excess SFA in the worm phospholipids that is almost always lethal to *paqr-2* mutants (**Fig 4A and 4B** and **S4A Fig**). Interestingly, evaluating the simpler palmitic acid (PA; 16:0)/cis-vaccenic acid (CVC; 18:1 delta 11) ratio was equally predictive of lethality (**Fig 4A**). The only exception was the pfkA *E*. *coli* mutant grown on glucose that supported the growth of *paqr-2* mutant worms in spite of an elevated PA/CVC ratio (**S4A Fig**); this pfkA *E*. *coli* mutant had a very unusual glossy appearance indicative of complex metabolic reprogramming (see **Fig 3C**).

**Fig 5:**
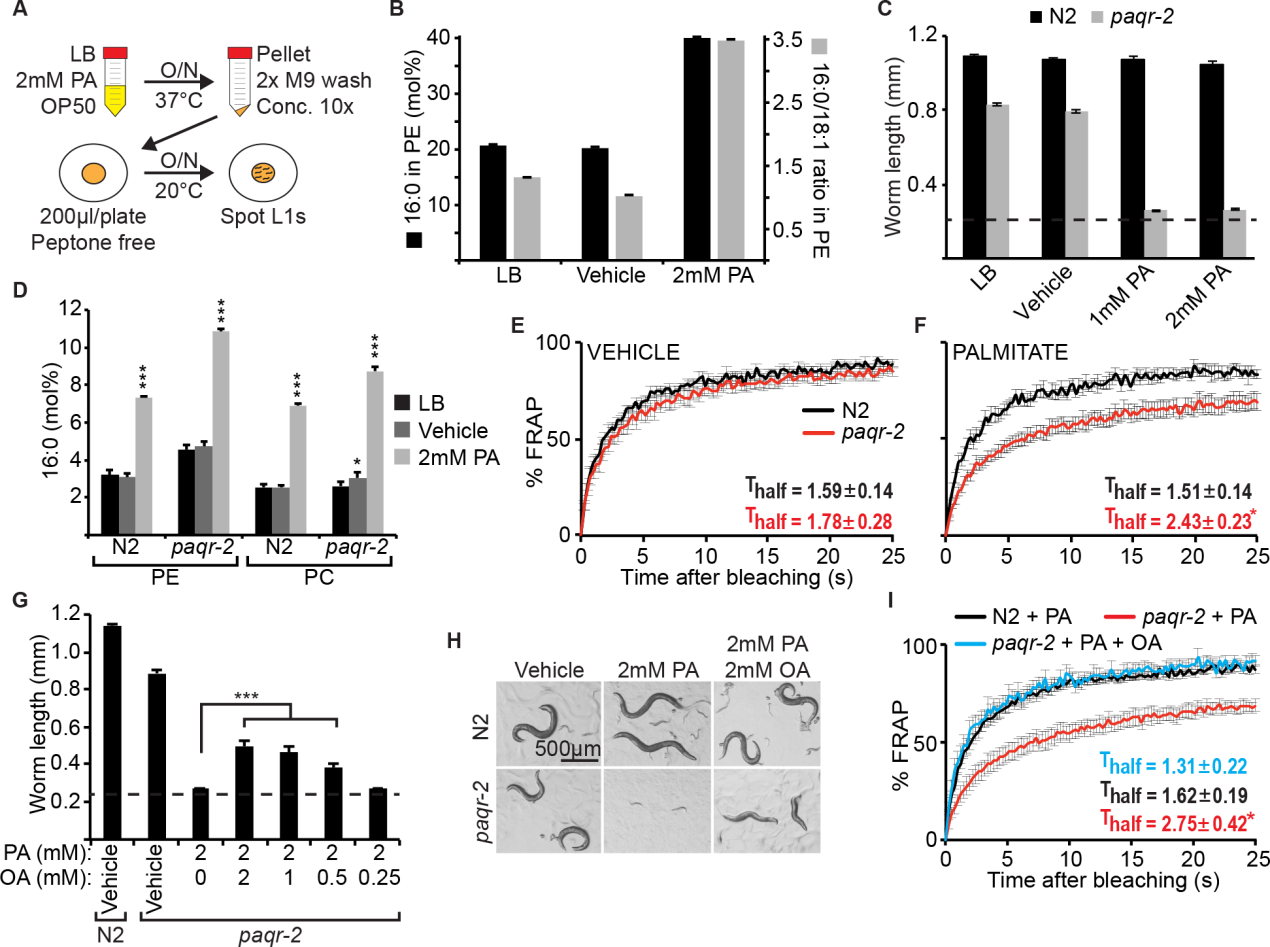
Palmitic acid causes membrane rigidity in the paqr-2 mutant. **(A)** Outline of the experimental design. **(B)** PA content and PA/OA ratio among the PEs of *E*. *coli* grown with or without PA; vehicle is ethanol. **(C)** *E*. *coli* grown pre-loaded with PA inhibits the growth of the *paqr-2* mutant but not of wild-type N2 worms. **(D)** PA content among the PEs and PCs of worms fed control or PA-loaded *E*. *coli*. Note that the *paqr-2* mutant accumulates more PA than wild-type N2 worms **(E-F)** FRAP of N2 and *paqr-2* mutants on normal plates or plates seeded with PA-loaded *E*. *coli*, respectively. Note the loss of membrane fluidity in *paqr-2* worms fed PA-loaded *E*. *coli*. **(G)** The growth of *paqr-2* is greatly ameliorated by pre-loading *E*. *coli* with both PA and OA rather than PA alone, with photographed worms shown in **(H)**. **(I)** FRAP showing that the rigidifying effects of PA pre-loaded dietary *E*. *coli* on the *paqr-2* mutant are abrogated by pre-loading the *E coli* with both PA and OA. The dashed line in **(C)** and **(G)** represents the approximate length of the L1s at the start of the experiments.

To test directly the effect of SFAs, we developed a protocol to pre-load *E*. *coli* with the SFA PA (**Fig 5A**), resulting in a doubling of dietary PA content **(Fig 5B)**. Using this method, we found that ≥1 mM PA during the cultivation of the *E*. *coli* produces a diet that is extremely toxic to the *paqr-2* mutant but harmless to wild-type worms (**Fig 5C**). This toxicity is accompanied by an increase in PA among phospholipids in the worms that is more pronounced in *paqr-2* mutants **(Fig 5D)**, and confers a dramatic decrease in membrane fluidity in the *paqr-2* mutant (**Fig 5E and 5F**). Consistently, wild-type worms respond to PA-loaded *E*. *coli* by strongly upregulating a GFP-reporter of the Δ9-desaturase FAT-7, which the *paqr-2* mutant fails to do (**S5 Fig)**. *paqr-2* is therefore required for the upregulation of desaturases that prevent membrane rigidification by dietary SFAs. Importantly, preloading the bacterial diet with a combination of PA and oleic acid (OA; 18:1 delta 9), and thus re-setting the 16:0/18:1 ratio **(S4B Fig)**, greatly reduces toxicity and rigidification of the membrane in *paqr-2* mutants (**Fig 5G–5I**).

### Mammalian AdipoR2 counters membrane rigidification by exogenous SFAs

**Fig 6:**
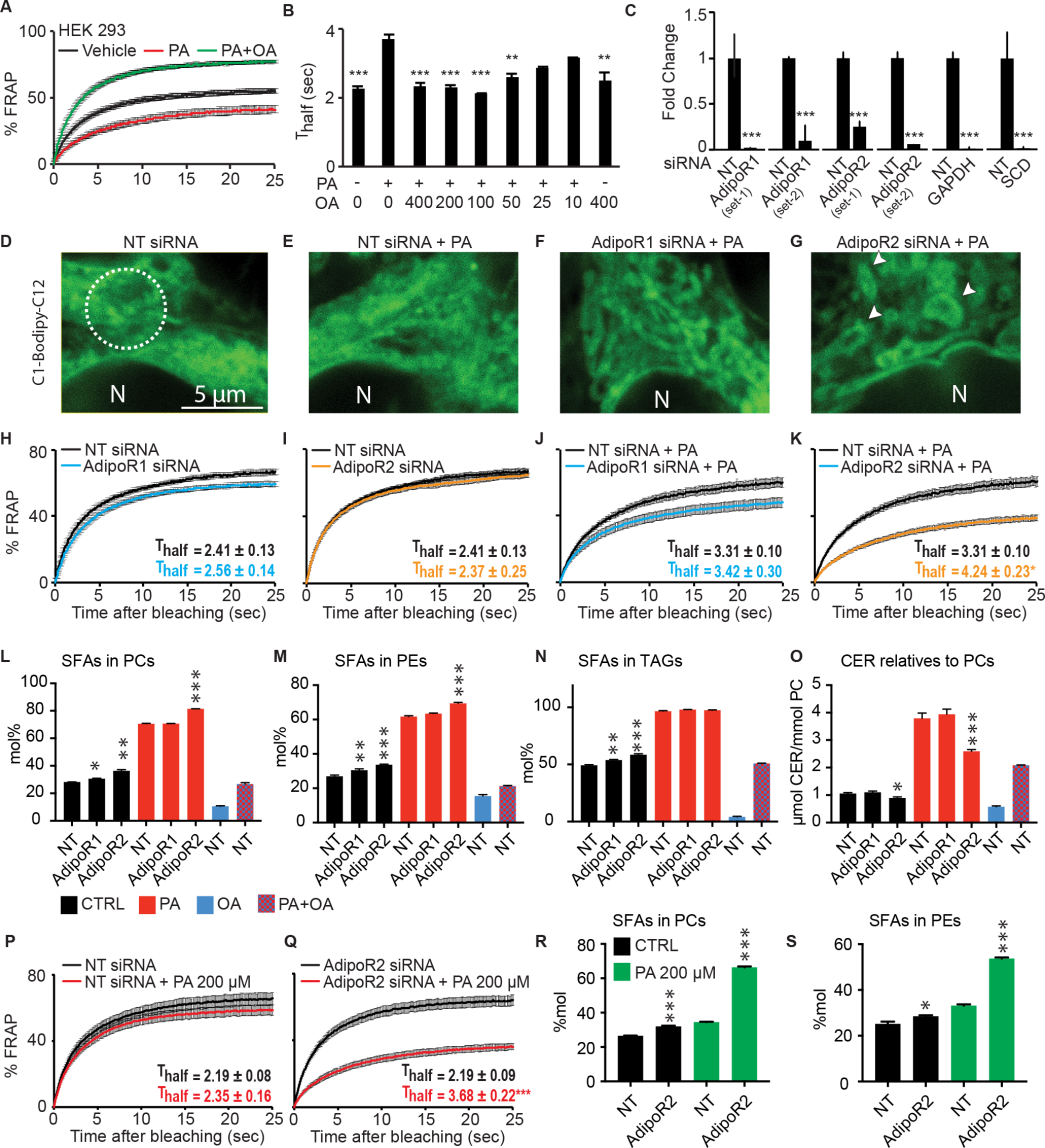
Mammalian AdipoR2 prevents rigidification by palmitic acid. **(A)** FRAP analysis showing that 400 μM PA causes reduced membrane fluidity in HEK293 cells and that 400 μM OA causes increased membrane fluidity even when PA is included. **(B)** T_half_ values from FRAP analyses performed with different concentrations of PA and OA. **(C)** Quantitative PCR results showing the degree of siRNA inhibition of the indicated genes. **(D-G)** The morphology of HEK293 cells is altered by PA when AdipoR2 is knocked down. Note the presence of numerous circular structures in the BODIPY-labeled cells treated with AdipoR2 siRNA (arrows). Nuclei are indicated by the letter "N", and the circle in **(D)** indicates the size of the area bleached in FRAP experiments with HEK293 cells. **(H-K)** FRAP analyses showing that AdipoR2 knockdown greatly increases the rigidifying effect of PA. **(L-O)** Lipidomics analysis of HEK 293 cells cultivated 24 hours in serum free media or serum-free media containing PA, OA or PA + OA and treated with non-target siRNA (NT) or AdipoR1 or AdipoR2 siRNA, as indicated. Note that PA alone causes a dramatic increase in SFAs among PCs, PEs and TAGs, and that this effect is increased by AdipoR2 knockdown. **(P-Q**) FRAP analyses showing that AdipoR2 knockdown greatly increases the rigidifying effect of 200 μM PA. **(R-S)** Lipidomics analysis showing that AdipoR2 siRNA also causes an excess of SFAs among PCs and PEs when the cells are incubated with 200 μM PA. Statistical analysis in **L-O** and **R-S** were done by comparing siRNA-treated cells with the non-target siRNA cultivated under the same conditions.

Others have shown that the addition of PA to mammalian cells causes membrane rigidification [16–18]. We were able to verify this in human HEK293 cells using the FRAP assay, and also found that the inclusion of OA counters the rigidifying effects of PA (**Fig 6A and 6B** and **Table 1**). To test whether the AdipoRs provide protection against the rigidifying effects of PA, we optimized conditions to knockdown the levels of the AdipoRs and other genes using siRNA (**Fig 6C**). AdipoR2 knockdown (but not AdipoR1 knockdown) caused a clear disorganization of the cellular appearance when HEK293 cells are treated with PA, suggesting a toxic effect (**Fig 6D–6G** and **S6A–S6D Fig**). Inhibition of AdipoR1 or AdipoR2 using two independent sets of siRNA oligonucleotides had no effects on the membrane fluidity of HEK293 cells grown under normal conditions (**Fig 6H and 6I; S6E and S6F Fig)**. In contrast, AdipoR2 knockdown (but again not AdipoR1 knockdown) caused a dramatic increase in membrane rigidification of PA-treated HEK293 cells (**Fig 6J and 6K; S6G and S6H Fig**). As controls, inhibition of the non-essential gene GAPDH had no effect on membrane fluidity, while inhibition of SCD, encoding a stearyl-CoA desaturase, caused the expected reduction of membrane fluidity when HEK293 cells are challenged with PA (**S6I and S6L Fig**). Additionally, FCCP, an uncoupler of mitochondrial oxidative phosphorylation, did not affect membrane fluidity; this demonstrates that toxicity in itself is not sufficient to lower membrane fluidity (**Table 1**), as others have also shown [19]. To summarize, among all our FRAP experiments only AdipoR2 and SCD knockdown enhanced the rigidifying effects of PA (**Table 1**).

**Table 1:** T_half_ values from FRAP experiments in HEK293 cells.

No	Sample	T_half_ ± SEM	*p* value
1	NT siRNA	2.26 ± 0.13	
	NT siRNA + 400 μM PA	3.64 ± 0.18	0.0000
2	NT siRNA	2.41 ± 0.13	
	AdipoR1 siRNA (set 1)	2.56 ± 0.14	0.4462
	AdipoR2 siRNA (set 1)	2.37 ± 0.25	0.8971
3	NT siRNA + 400 μM PA	3.31 ± 0.10	
	AdipoR1 siRNA (set 1) + 400 μM PA	3.42 ± 0.30	0.8530
	AdipoR2 siRNA (set 1) + 400 μM PA	4.24 ± 0.23	0.0275
4	NT siRNA	2.19 ± 0.10	
	AdipoR1 siRNA (set 2)	2.21 ± 0.06	0.8671
	AdipoR2 siRNA (set 2)	2.06 ± 0.11	0.4147
5	NT siRNA + 400 μM PA	3.30 ± 0.60	
	AdipoR1 siRNA (set 2) + 400 μM PA	2.97 ± 0.30	0.4144
	AdipoR2 siRNA (set 2) + 400 μM PA	4.05 ± 0.21	0.0353
6	NT siRNA	2.19 ± 0.08	
	NT siRNA + 200 μM PA	2.35 ± 0.35	0.4647
7	AdipoR2 siRNA (set 1)	2.19 ± 0.09	
	AdipoR2 siRNA (set 1) + 200 μM PA	3.68 ± 0.22	0.0002
8	AdipoR2 siRNA (set 2)	2.07 ± 0.13	
	AdipoR2 siRNA (set 2) + 200 μM PA	3.57 ± 0.23	0.0003
9	NT siRNA	2.32 ± 0.05	
	GAPDH siRNA	2.33 ± 0.05	0.9652
	SCD siRNA	2.56 ± 0.08	0.0912
10	NT siRNA + 400 μM PA	3.37 ± 0.25	
	GAPDH siRNA + 400 μM PA	3.20 ± 0.36	0.7169
11	NT siRNA + 400 μM PA	3.25 ± 0.11	
	SCD siRNA + 400 μM PA	3.90 ± 0.13	0.0106
12	Vehicle	2.47 ± 0.12	
	10 μM FCCP	2.37 ± 0.29	0.5394

As mentioned earlier, lipidomics analysis in *C*. *elegans* revealed that PAQR-2 is essential to keep phospholipid SFAs below a critical threshold when the worms are fed a SFA-rich diet. We found that a similar phenomenon occurs in HEK293 cells: there is a dramatic increase in the SFA content among PCs, PEs and TAGs when HEK293 cells are incubated in the presence of PA, and this effect is exacerbated when AdipoR2 is knocked-down using siRNA (**Fig 6L–6N**). Note that AdipoR2 siRNA causes an increased SFA content in PCs, PEs and TAGs even when PA is not added but that the effect is then more modest. Given the proposed ceramidase activity of AdipoR2 [3,8], we also examined the levels of ceramides in our experiments. PA is a precursor for the synthesis of ceramides and we were therefore not surprised that their relative levels were increased in PA-treated cells (**Fig 6O**). However, the ceramide levels in PA-treated cells did not increase as much when AdipoR2 had been knocked-down by siRNA, which is somewhat surprising if AdipoR2 acts as a ceramidase (**Fig 6O**).

Up to this point, our experiments using PA employed concentrations of 400 μM, which is the concentration at which a detectable rigidifying effect occurs on normal cells. However, we reasoned that if AdipoR2 acts by preventing membrane rigidification, then cells where AdipoR2 has been knocked-down using siRNA should be sensitive to lower amounts of PA. This is indeed the case: 200 μM PA causes a dramatic decrease in membrane fluidity in AdipoR2 siRNA-treated cells but not in control cells (**Fig 6P and 6Q**), and this rigidification is accompanied by an equally dramatic increase in the SFA content in both PCs and PEs (**Fig 6R and 6S**). Altogether, these results demonstrate that AdipoR2, like its *C*. *elegans* homolog PAQR-2, is required to prevent membrane rigidification by exogenous SFAs.

## Discussion

**Fig 7:**
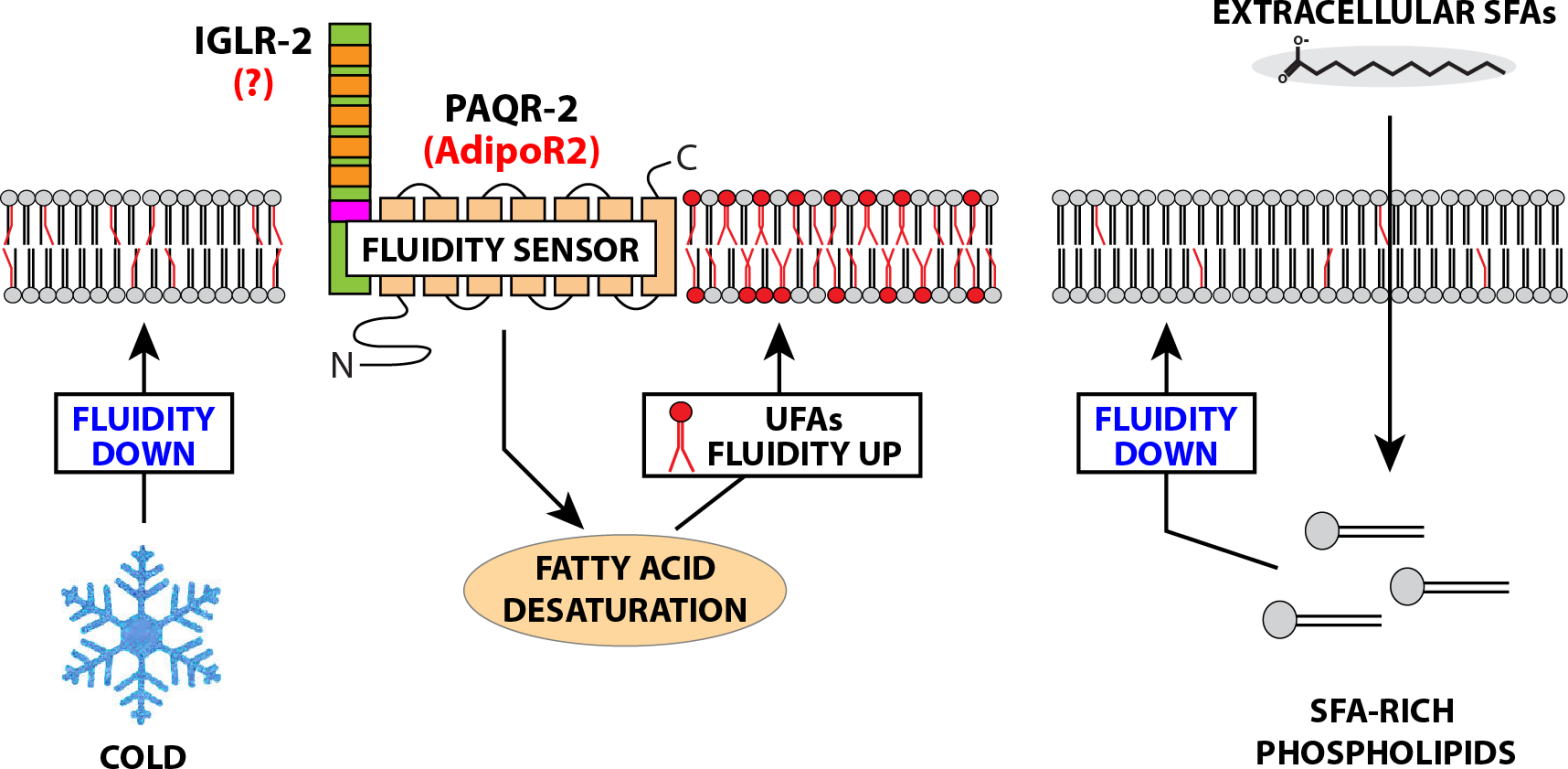
Model of membrane fluidity regulation by PAQR-2/AdipoR2. This revised model proposes that PAQR-2 (AdipoR2 in mammals) and IGLR-2 (homolog unknown in human) act as sensors that are activated by low membrane fluidity caused by cold (in *C*. *elegans*) or exogenous SFAs (in *C*. *elegans* and human cells), and act on downstream effectors to restore fluidity by promoting changes in fatty acid metabolism, including promoting the activity of fatty acid desaturases. In this visualization, the red head groups indicate phospholipids with UFAs resulting from PAQR-2/AdipoR2 activity.

The fatty acid composition of cellular membranes reflects the composition of the dietary fats. This is especially evident for complex dietary PUFAs that become incorporated into membrane phospholipids in *C*. *elegans* [20] and in mammals [21–23]. However, regulatory mechanisms must exist to adjust membrane composition, hence properties, in response to diets with a wide range of SFA/UFA ratios. The present study shows that PAQR-2 in *C*. *elegans*, and its homolog AdipoR2 in mammals, are essential to maintain membrane homeostasis in the presence of exogenous SFAs (see the model in **Fig 7**).

*De novo* lipogenesis inside the worm is likely adjusted to cellular needs, whereby desaturation is coordinated with lipogenesis to produce a healthy mixture of SFAs and UFAs [24], which is also the case in mammalian cells [25]. However, the worm cannot control the nature of external factors that could insult the membranes, such as temperature or dietary fat composition. PAQR-2 seems especially required in response to such external factors. There is a remarkably high turnover of membranes in *C*. *elegans*: nearly 70% of phospholipids are renewed daily in post-reproductive adults, with a majority of recruited FAs being of dietary origin [20]. Similarly, *C*. *elegans* fat stores also reflects the fatty acid composition of dietary bacteria [26]. Such high reliance on dietary FAs necessitates mechanisms to sense and adjust lipid composition to fit cellular needs, and PAQR-2 seems to act as such a sensor/regulator. The desaturases *fat-5*, *-6*, and *-7* are key regulators of membrane turnover and composition in *C*. *elegans* [20,27], and their regulation by PAQR-2 is therefore a potent mechanism to regulate membrane homeostasis [10,11].

PAQR-2 is not the only regulator of membrane homeostasis identified in eukaryotes. In particular, changes in the lipid composition of the endoplasmic reticulum (ER), where lipids are synthesized, can cause the induction of the unfolded protein response in yeast and mammals, which regulates several genes involved in lipid biosynthesis [28,29]. In the ER, it is the protein Ire1 that senses aberrant properties of the membrane via an amphipathic helix [30]. The mechanism of membrane stress sensing by PAQR-2 has not been identified, but it is possible that its conformation or interaction with IGLR-2, an obligate partner for PAQR-2 activity, is sensitive to the plasma membrane environment where PAQR-2 is localized [11].

Besides influencing membrane properties, lipid composition is important for many other aspects of *C*. *elegans* biology. For example, PC levels regulate SBP-1 (a homolog of mammalian SREBP) by altering membrane properties important for vesicular trafficking [31,32], and specific UFAs regulate germline and other developmental processes [33,34]. It is therefore not surprising that lipid disequilibrium causes activation of stress responses such as the endoplasmic reticulum unfolded protein response [35,36]. By acting as a regulator of lipid metabolism, PAQR-2 is therefore likely to impinge on many other processes besides membrane homeostasis.

Our study of membrane composition and fluidity in human HEK293 cells led to several intriguing observations. We noted for example that while AdipoR2 siRNA strongly aggravated the effects of PA on membrane composition and fluidity, AdipoR1 siRNA had no significant effect. This suggests that, at least in this cell line, AdipoR2 has a greater role in membrane homeostasis than AdipoR1, a situation reminiscent of *C*. *elegans* where PAQR-2 is more important than PAQR-1 [9]. Our work also suggests that AdipoR2 can regulate membrane fluidity in the absence of supplemented adiponectin, its proposed ligand secreted nearly exclusively by adipocytes [37–39], since the siRNA-treated HEK293 cells were cultivated for 24 hours in serum-free media prior to FRAP analysis. Again, this is analogous to the situation in *C*. *elegans*, where no adiponectin homolog has been identified either by sequence homology searches or in a screen for *paqr-2* genocopiers [11]. In this context, it is important to note that Vasiliauskaité-Brooks and co-workers have shown that the AdipoRs ceramidase activity is not strictly adiponectin dependent [3]. Finally, we found that AdipoR2 siRNA led to a decrease in the abundance of ceramides, which is counter-intuitive if AdipoR2 is a ceramidase as has been proposed [3,6,8]. Further studies will hopefully clarify this and other observations.

Mammalian mutant phenotypes suggest that the AdipoRs have an evolutionary conserved role in preventing dietary FA toxicity. In particular, mouse mutants lacking adiponectin, the ligand for AdipoR1 and AdipoR2, develop metabolic complications (glucose intolerance, insulin resistance) only when fed a high fat diet [40], which is analogous to the *C*. *elegans paqr-2* mutants that show severe phenotypes when fed a SFA-rich diet. Also like the *C*. *elegans paqr-*2 mutant, AdipoR1 and AdipoR2 knockout mice show several defects in lipid homeostasis [41–44]. Conversely, overexpression of the AdipoRs leads to improved lipid homeostasis and can even rescue the diabetic phenotype in mice lacking a functional leptin receptor [7]. In human, a rare loss-of-function mutation in AdipoR1 causes autosomal dominant retinitis pigmentosa [45], which is likely due to AdipoR1 regulating fatty acid composition of the retina [46]. In the future, it will be interesting to investigate further the relevance of our findings for human physiology. In particular, several studies have documented an increase in both membrane SFAs and rigidity in diabetics [12,47–55], and these changes may result from the conversion of glucose into SFAs via *de novo* lipogenesis. Membrane rigidification could therefore be a component of glucose toxicity, and the oft-documented anti-diabetic activities of the AdipoRs could lie in their ability to counter such a rigidifying effect.

## Materials and methods

### *C*. *elegans* strains and cultivation

The wild-type *C*. *elegans* reference strain N2 and the mutant alleles studied are available from the *C*. *elegans* Genetics Center (CGC; MN; USA). The *pfat-7*::*GFP (rtIs30)* carrying strain HA1842 was a kind gift from Amy Walker [31], and its quantification was performed as previously described [10]. *C*. *elegans* strains maintenance and experiments were performed at 20°C using the *E*. *coli* strain OP50 as food source, which was maintained on LB plates kept at 4°C (re-streaked every 6–8 weeks) and single colonies were picked for overnight cultivation at 37°C in LB medium then used to seed NGM plates [56]; new LB plates were streaked every 3–4 months from OP50 stocks kept frozen at -80°C.

### Plates with supplements

Stock solutions of supplements (1M glucose, dihydroxyacetone, pyruvate, and lactate; 5 M NaCl and KCl; 50 mM fluvastatin) were filter sterilized then added to cooled NGM after autoclaving; 100 mM paraquat, 30 mM FCCP (in ethanol), pure DMSO and pure glycerol were used without sterilization.

### Seeding of NGM plates with *E*. *coli* mutants

The Keio collection of *E*. *coli* K-12 in-frame, single-gene knockouts was used as source of *E*. *coli* mutants and kept in the presence of 50 μg/ml kanamycin [15]. Mutants were picked as single colonies from LB plates and cultivated overnight at 37°C in LB then seeded onto NGM plates with or without additives. All mutants were confirmed by PCR.

### MacConkey agar assay

*E*. *coli* strains were streaked onto MacConkey agar plates with 0.4% glucose, grown at 37°C over night and scored for colony color.

### Growth and tail tip scoring assays

For length measurement studies, synchronized L1s were plated onto test plates seeded with *E*. *coli*, and worms were mounted then photographed 96 hour (oleic acid rescue experiments) or 72 hours (all other experiments) later. The length of >20 worms was measured using ImageJ [57]. Quantification of the withered tail tip phenotype was done on synchronous 1-day old adult populations, i.e. 72 h post L1 (n≥100) [10].

### Fluorescence recovery after photobleaching (FRAP)

FRAP experiments in *C*. *elegans* were carried out using a membrane-associated prenylated GFP reporter expressed in intestinal cells, as previously described and using a Zeiss LSM700inv laser scanning confocal microscope with a 40X water immersion objective [11,58]. Briefly, the GFP-positive membranes were photobleached over a circular (7 pixel radius) using 20 iterations of the 488 nm laser with 50% laser power transmission. Images were collected at a 12-bit intensity resolution over 256x256 pixels (digital zoom 4X) using a pixel dwell time of 1.58 μsec, and were all acquired under identical settings. For FRAP in mammalian cells, HEK293 cells were stained with BODIPY 500/510 C_1_, C_12_ (4,4-Difluoro-5-Methyl-4-Bora-3a,4a-Diaza-*s*-Indacene-3-Dodecanoic Acid) (Invitrogen) at 2 μg/ml in PBS for 10 min at 37°C. FRAP images were acquired with an LSM880 confocal microscope equipped with a live cell chamber (set at 37°C and 5% CO_2_) and ZEN software (Zeiss) with a 40X water immersion objective. Cells were excited with a 488 nm laser and the emission between 493 and 589 nm recorded. Images were acquired with 16 bits image depth and 256x256 resolution using a pixel dwell of ~ 1.34 μs. Ten pre-bleaching images were collected and then the region of interest was beached with 50% of laser power. The recovery of fluorescence was traced for 25 seconds. Fluorescence recovery and T_half_ were calculated as previously described [11].

### Pre-loading of *E*. *coli* with fatty acids

Stocks of 0.1 M palmitic acid or 0.5 M oleic acid dissolved in ethanol were diluted in LB media to final concentrations of 0.25–2 mM, inoculated with OP50 bacteria, then shaken overnight at 37°C. The bacteria were then washed twice with M9 to remove fatty acids and growth media, diluted to equal OD_600_, concentrated 10X by centrifugation, dissolved in M9 and seeded onto NGM plates lacking peptone (200μl/plate). Worms were added the following day.

### Lipidomics

For worm lipidomics, samples were composed of synchronized L4 larvae (one 9 cm diameter plate/sample) grown overnight on OP50-seeded NGM, NGM containing 20mM glucose, 0.5% glycerol, 20 mM pyruvate, or plates lacking peptone but seeded with fatty acid-supplemented bacteria. Worms were washed 3 times with M9, pelleted and stored at -80°C until analysis. For bacterial lipidomics, *E*. *coli* liquid cultures grown overnight at 37°C were seeded on NGM, NGM containing 20 mM glucose, 0.5% glycerol, 20 mM pyruvate or concentrated (as described above) and seeded on plates lacking peptone for lipid-supplemented cultures, kept upside-down at 20°C then washed off 96 h later using pure water, pelleted then frozen at -80°C until analysis. For HEK293 lipidomics, cells were cultivated in serum-free media with or without fatty acids for 24 h prior to harvesting using TrypLE Express (Gibco). For lipid extraction, the pellet was sonicated for 10 minutes in methanol and then extracted according to published methods [59]. Internal standards were added during the extraction. Lipid extracts were evaporated and reconstituted in chloroform:methanol [1:2] with 5 mM ammonium acetate. This solution was infused directly (shotgun approach) into a QTRAP 5500 mass spectrophotometer (Sciex, Toronto, Canada) equipped with a Nanomate Triversa (Advion Bioscience, Ithaca, NY) as described previously [60]. Phospholipids were measured using multiple precursor ion scanning [61,62]. Ceramides from HEK293 cells were measured using ultra performance liquid chromatography coupled to tandem mass spectrometry according to previous publication [63]. The data was evaluated using the LipidView software (Sciex, Toronto, Canada). The complete lipidomics dataset is provided in the supplementary **S1 File**.

### Cultivation of HEK293

HEK293 were grown in DMEM containing glucose 1 g/l, pyruvate and GlutaMAX and supplemented with 10% fetal bovine serum, 1% non-essential amino acids, HEPES 10 mM and 1% penicillin and streptomycin (all from Life Technologies) at 37°C in a water humidified 5% CO_2_ incubator. Cells were sub-cultured twice a week at 90% confluence. Cells were cultivated on treated plastic flask and multi-dish plates (Nunc). For FRAP experiments, HEK293 were seeded in glass bottom dishes (Ibidi) pre-coated with 0.1% porcine gelatin (Sigma).

### siRNA in HEK293 cells

The following pre-designed siRNAs were purchased from Dharmacon: AdipoR1 J-007800-10-005 (set 1) and J-007800-09-0002 (set 2), AdipoR2 J-007801-10-0005 (set 1) and J-007801-09-0002 (set 2), GAPDH D-001830-10-05, Non-target D-001810-01-05 or D-001810-10-05, and SCD J-005061-07-0005. Transfection of 25 nM siRNA was performed in complete media using Viromer Blue according to the manufacturer’s instructions 1X (Lipocalyx). Knockdown gene expression was verified 48 h after transfection.

### Quantitative PCR in HEK293 cells

Total cellular RNA was isolated using RNeasy Kit according to the manufacturer’s instructions (Qiagen) and quantified using a NanoDrop spectrophotometer (ND-1000; Thermo Scientific). cDNA was obtained using a High Capacity cDNA Reverse Transcription Kit (Applied Biosystem) with random hexamers. qPCR were performed with a CFX Connect thermal cycler (Bio Rad) using Hot FIREpol EvaGreen qPCR SuperMix (Solis Biodyne) and standard primers. Samples were measured as triplicates. The relative expression of each gene was calculated according to the ΔΔCT method [64]. Expression of the housekeeping gene PPIA was used to normalize for variations in RNA input. Primers used were: AdipoR1-For (CCATCTGCTTGGTTTCGTGC) and -Rev (AGACGGTGTGAAAGAGCCAG), AdipoR2-For (TCATCTGTGTGCTGGGCATT) and -Rev (CTATCTGCCCTATGGTGGCG), GAPDH-For (GAGAAGGCTGGGGCTCATTT) and -Rev (TAAGCAGTTGGTGGTGCAGG), PPIA-For (GTCTCCTTTGAGCTGTTTGCAG) and -Rev (GGACAAGATGCCAGGACCC), and SCD-For (TTCGTTGCCACTTTCTTGCG) and -Rev (TGGTGGTAGTTGTGGAAGCC).

### HEK293 fatty acid and FCCP treatment

PA and OA were dissolved in sterile DMSO (Sigma) then mixed with fatty acid-free BSA (Sigma) in serum-free medium for 20 min at room temperature. The molecular ratio of BSA to fatty acid was 1 to 5.3 (except in the experiment using using 200 μM PA in which case the ratio was 1 to 2.65). Cells were then cultivated in this serum-free media containing the fatty acids for 24 h prior to analysis, and with 400 μM PA or OA being used unless stated otherwise. FCCP was dissolved in ethanol to produce a 30 mM stock and used at a concentration of 10 μM in serum-free-media for 4 hours prior to analysis.

### Statistics

Error bars for worm length measurements show the standard error of the mean, and *t-*tests were used to identify significant differences between worm lengths. SFA/MUFA ratios were normalized using a *log*_*N*_ conversion prior to test for significance using a *t-*test. Error bars for the frequency of the tail tip defect show the 95% confidence interval and significant differences determined using *Z-tests*. *t-*tests were also used to determine significance in FRAP experiments, and the lipidomics data in HEK293 cells was analyzed using ANOVA and a Dunnett's multiple comparisons test. All experiments were repeated several times with similar results. Asterisks are used in the figures to indicate various degrees of significance, where *: *p*<0.05; **: *p*<0.01; and ***: *p*<0.001.

## Supporting information

S1 Fig The *paqr-2* mutant is not especially sensitive to other types of stressors. **(A-F)** Length of wild-type N2 and *paqr-2* mutant worms cultivated for 72 hours on various concentrations of stressors, with representative images shown in **(G)**. The dashed line in **(A-F)** represents the approximate length of the L1s at the start of the experiments.
(TIF)

S2 Fig Effect of the ΔPTS OP50 and BW25113 *E*. *coli* strains on *paqr-2*. **(A)** The ΔPTS mutation in the *E*. *coli* strain OP50 does not abolish the toxicity of glycerol or pyruvate for the *C*. *elegans paqr-2* mutant. **(B-C)** The ΔPTS OP50 and BW25113 *E*. *coli* strains do not prevent the cold intolerance and tail tip phenotypes of the *C*. *elegans paqr-2* mutant. **(D)** All five glycolysis-related metabolites tested are toxic to *paqr-2* mutants fed OP50 *E*. *coli* but only two (glucose and pyruvate) are toxic when *paqr-2* mutants are fed BW25113 *E*. *coli*. Note that the metabolites are added to the culture plates before seeding with *E*. *coli*. The dashed line in **(A, B and D)** represents the approximate length of the L1s at the start of the experiments.
(TIF)

S3 Fig Mutations in *E*. *coli* metabolic enzymes modify dietary toxicity in the *C*. *elegans paqr-2* mutant. The reference *E*. *coli* strains BW25113 and several single mutants from the Keio collection were tested for their ability to modify the growth of the *C*. *elegans paqr-2* mutant when provided as food on NGM plates **(A)**, NGM plates containing 20 mM glucose **(B)** or NGM plates containing 0.5% glycerol **(C)**. **(D)** Mutations affecting the pentose phosphate pathway in *E*. *coli* did not prevent the toxicity of glucose or glycerol in *paqr-2* mutant worms **(E)** Photographs of various *E*. *coli* strains grown on MacConkey agar; only strains capable of metabolizing glucose produce the red color. Note in particular that ΔPTS OP50, ptsG and pfkA are poor at metabolizing glucose. The dashed line in **(A-D)** represents the approximate length of the L1s at the start of the experiments.
(TIF)

S4 Fig Analysis of lipids in *E*. *coli*. **(A)** Ratio of PA/OA in the PEs of the control *E*. *coli* strain BW25113 and three mutants cultivated in the presence of 20 mM glucose or 0.5% glycerol. Orange bars indicate conditions that prevented growth and were lethal to *paqr-2* mutants. **(B)** Proportion of PA and OA, and PA/OA ratio, among the PEs of *E*. *coli* cultivated under control conditions (LB and vehicle) or pre-loaded with FAs. Note that inclusion of OA normalizes the amounts of PA and leads to a low PA/OA ratio.
(TIF)

S5 Fig The *paqr-2* mutant does not upregulate *pfat-7*::*GFP* in response to PA. **(A)** Photographs of *pfat-7*::*GFP* transgenic N2 or *paqr-*2 mutant worms grown on *E*. *coli* pre-loaded without or with 2 mM PA. **(B)** Quantification of the p*fat-7*::*GFP* fluorescence.
(TIF)

S6 Fig Additional HEK293 data. **(A-D)** The morphology of HEK293 cells is altered by PA when AdipoR2 is knocked down. Note the presence of numerous circular structures in the BODIPY-labeled cells treated with AdipoR2 siRNA. Nuclei are indicated by the letter "N", and the circle in **(A)** indicates the size of the area that would be bleached in a FRAP experiment. Yellow rectangles indicate the areas enlarged in **Fig 6**. **(E-L)** FRAP analysis in HEK293 cells comparing non-target siRNA with siRNA against various genes with or without PA.
(TIF)

S1 File Lipidomics dataset. This is an Excel file containing the complete numerical lipidomics data and organized as separate sheets for each lipidomics figure shown in the article.
(XLSX)
