# Supplementary figures and images for: The adiponectin receptor AdipoR2 and its Caenorhabditis elegans homolog PAQR-2 prevent membrane rigidification by exogenous saturated fatty acids
Source: PLoS Genet. 2017 Sep 8;13(9):e1007004. doi: 10.1371/journal.pgen.1007004 (PMC5607217; doi:10.1371/journal.pgen.1007004)

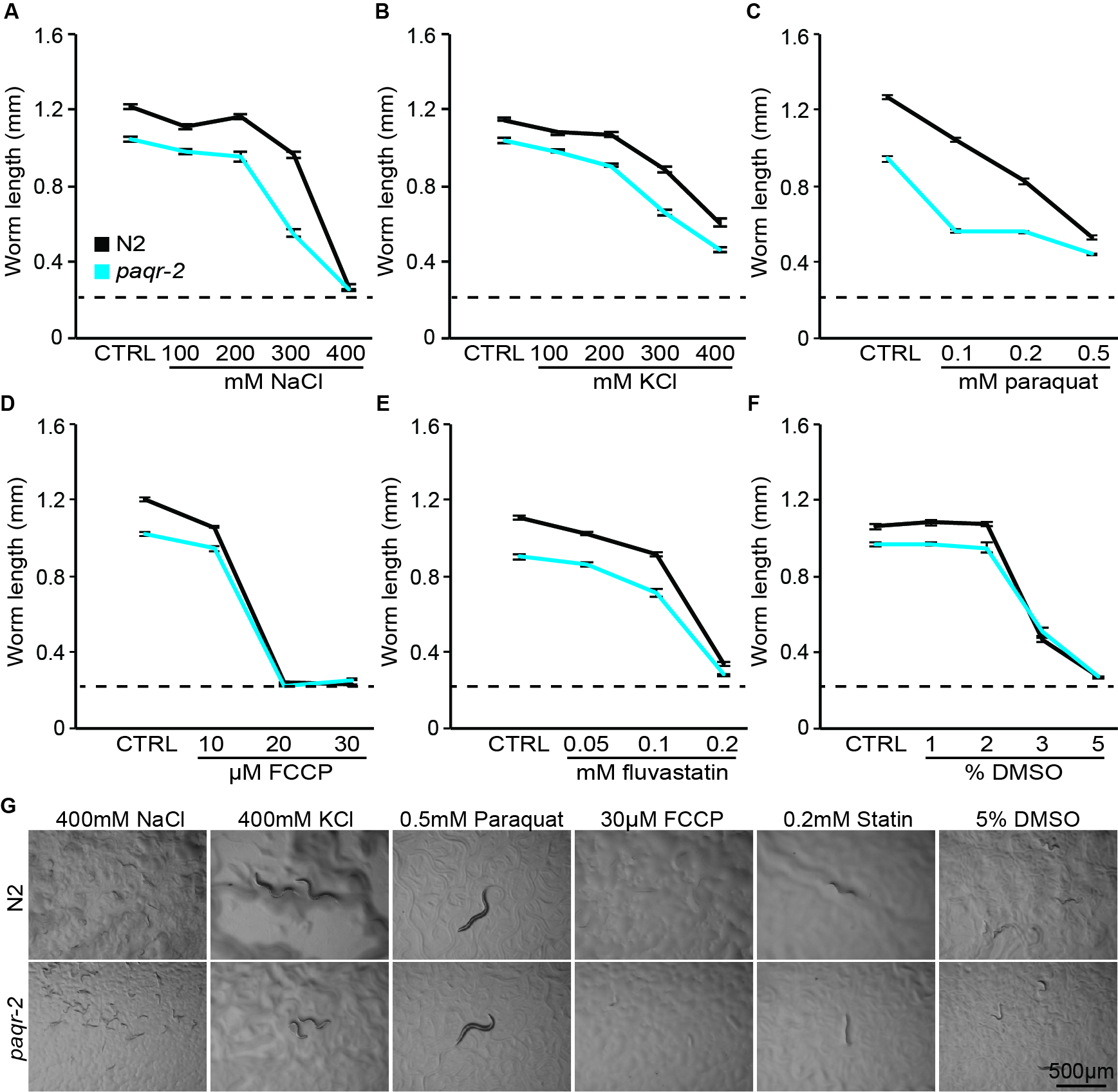

Supplement: S1 Fig — (A-F) Length of wild-type N2 and paqr-2 mutant worms cultivated for 72 hours on various concentrations of stressors, with representative images shown in (G). The dashed line in (A-F) represents the approximate length of the L1s at the start of the experiments. (TIF) [file pgen.1007004.s001.tif]

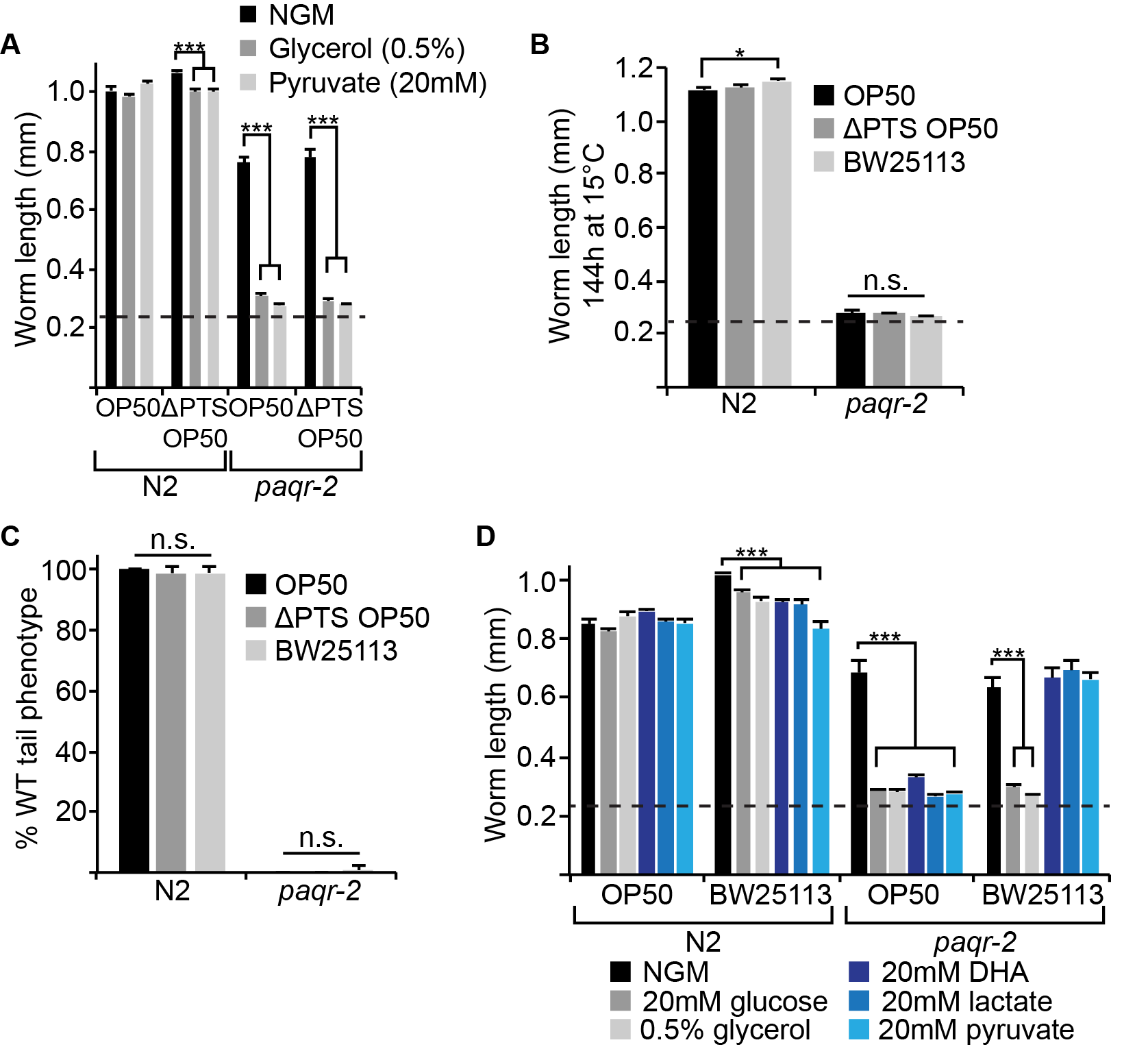

Supplement: S2 Fig — (A) The ΔPTS mutation in the E. coli strain OP50 does not abolish the toxicity of glycerol or pyruvate for the C. elegans paqr-2 mutant. (B-C) The ΔPTS OP50 and BW25113 E. coli strains do not prevent the cold intolerance and tail tip phenotypes of the C. elegans paqr-2 mutant. (D) All five glycolysis-related metabolites tested are toxic to paqr-2 mutants fed OP50 E. coli but only two (glucose and pyruvate) are toxic when paqr-2 mutants are fed BW25113 E. coli. Note that the metabolites are added to the culture plates before seeding with E. coli. The dashed line in (A, B and D) represents the approximate length of the L1s at the start of the experiments. (TIF) [file pgen.1007004.s002.tif]

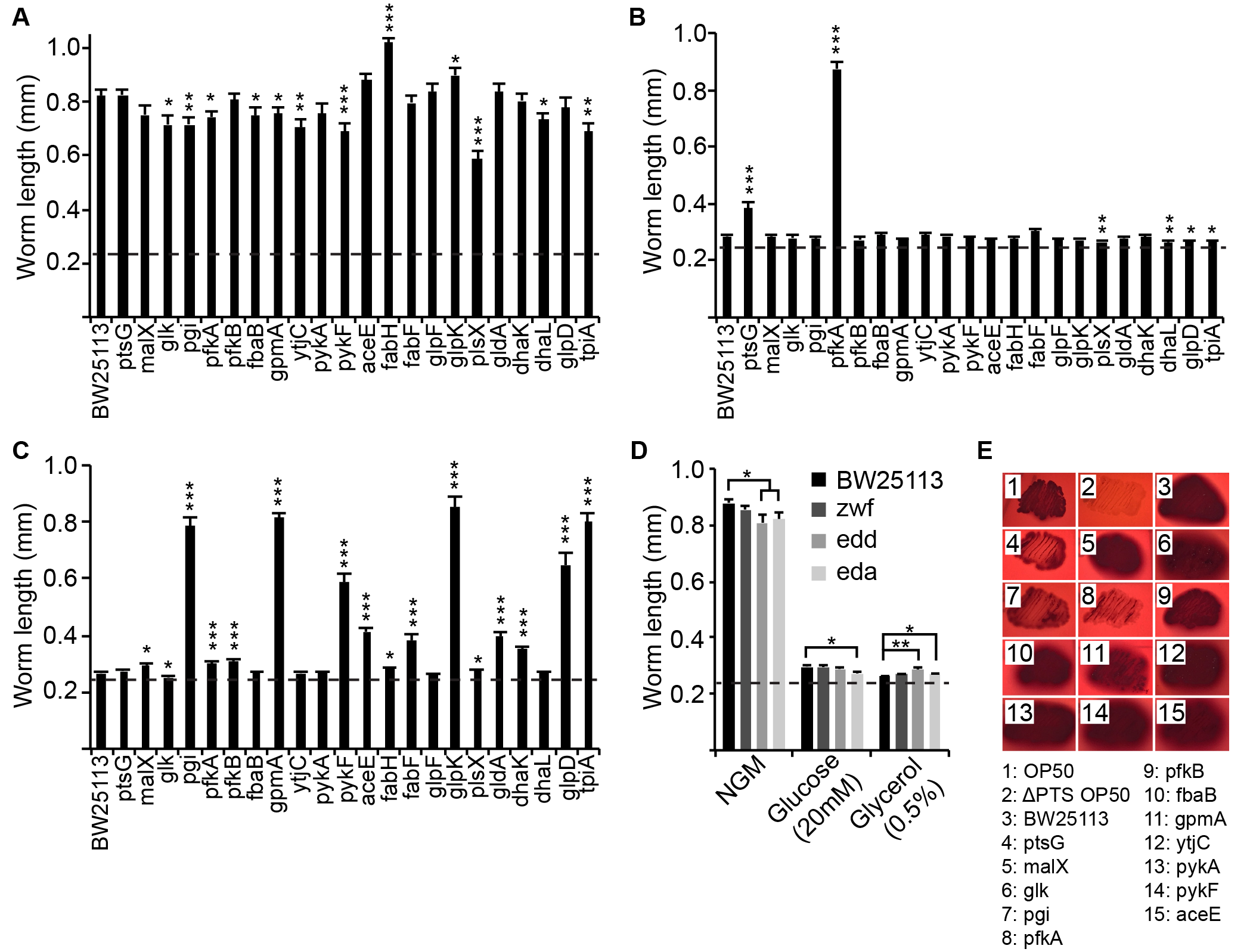

Supplement: S3 Fig — The reference E. coli strains BW25113 and several single mutants from the Keio collection were tested for their ability to modify the growth of the C. elegans paqr-2 mutant when provided as food on NGM plates (A), NGM plates containing 20 mM glucose (B) or NGM plates containing 0.5% glycerol (C). (D) Mutations affecting the pentose phosphate pathway in E. coli did not prevent the toxicity of glucose or glycerol in paqr-2 mutant worms (E) Photographs of various E. coli strains grown on MacConkey agar; only strains capable of metabolizing glucose produce the red color. Note in particular that ΔPTS OP50, ptsG and pfkA are poor at metabolizing glucose. The dashed line in (A-D) represents the approximate length of the L1s at the start of the experiments. (TIF) [file pgen.1007004.s003.tif]

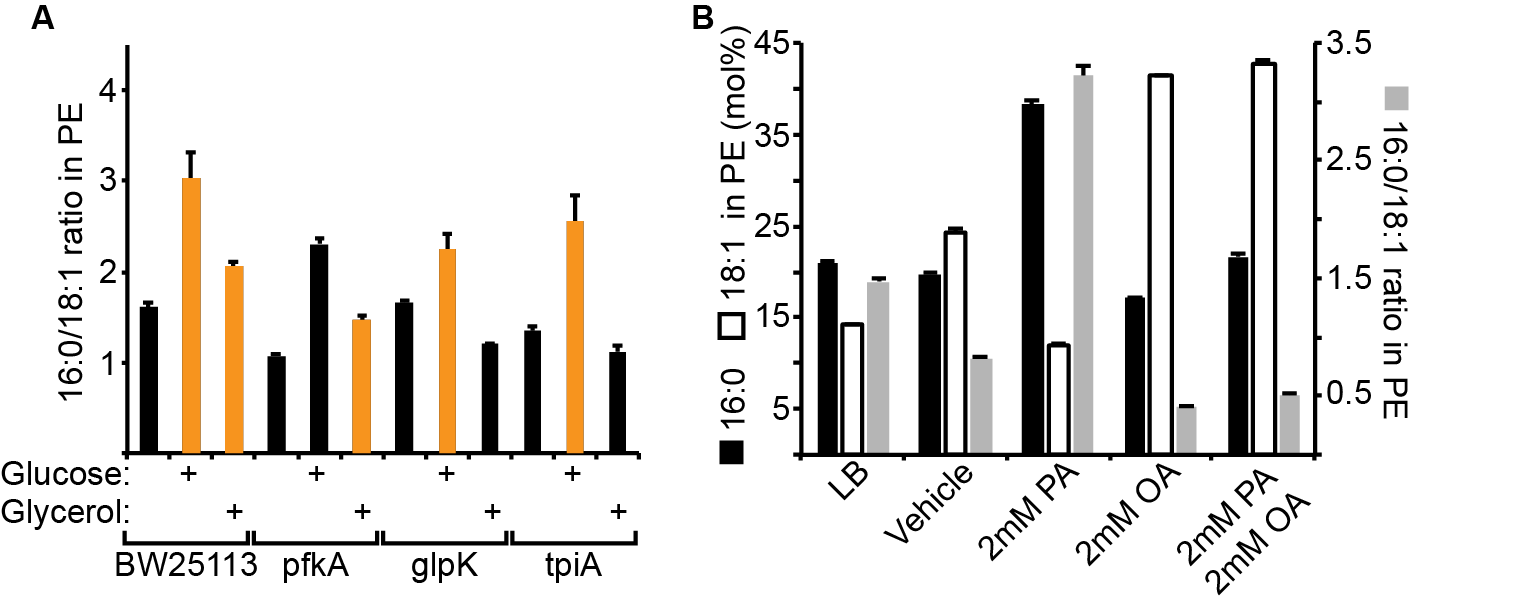

Supplement: S4 Fig — (A) Ratio of PA/OA in the PEs of the control E. coli strain BW25113 and three mutants cultivated in the presence of 20 mM glucose or 0.5% glycerol. Orange bars indicate conditions that prevented growth and were lethal to paqr-2 mutants. (B) Proportion of PA and OA, and PA/OA ratio, among the PEs of E. coli cultivated under control conditions (LB and vehicle) or pre-loaded with FAs. Note that inclusion of OA normalizes the amounts of PA and leads to a low PA/OA ratio. (TIF) [file pgen.1007004.s004.tif]

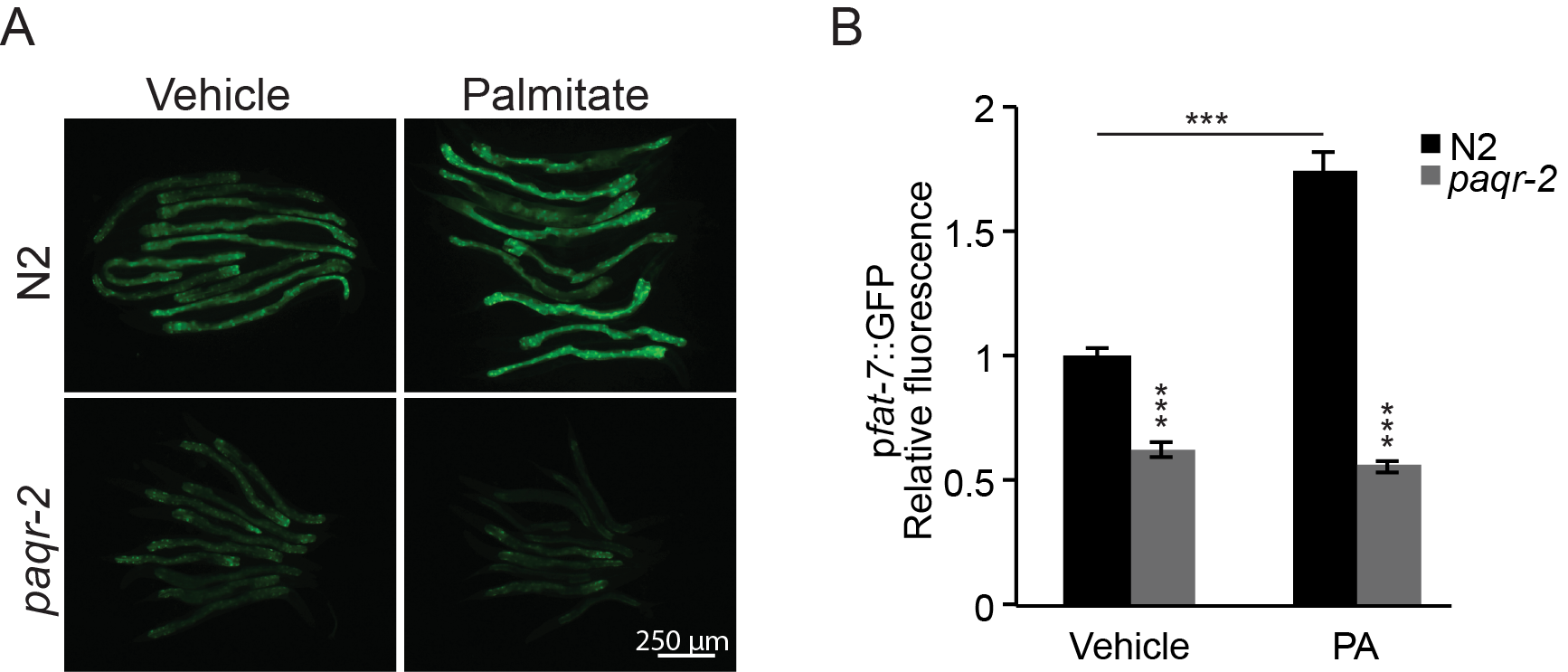

Supplement: S5 Fig — (A) Photographs of pfat-7::GFP transgenic N2 or paqr-2 mutant worms grown on E. coli pre-loaded without or with 2 mM PA. (B) Quantification of the pfat-7::GFP fluorescence. (TIF) [file pgen.1007004.s005.tif]

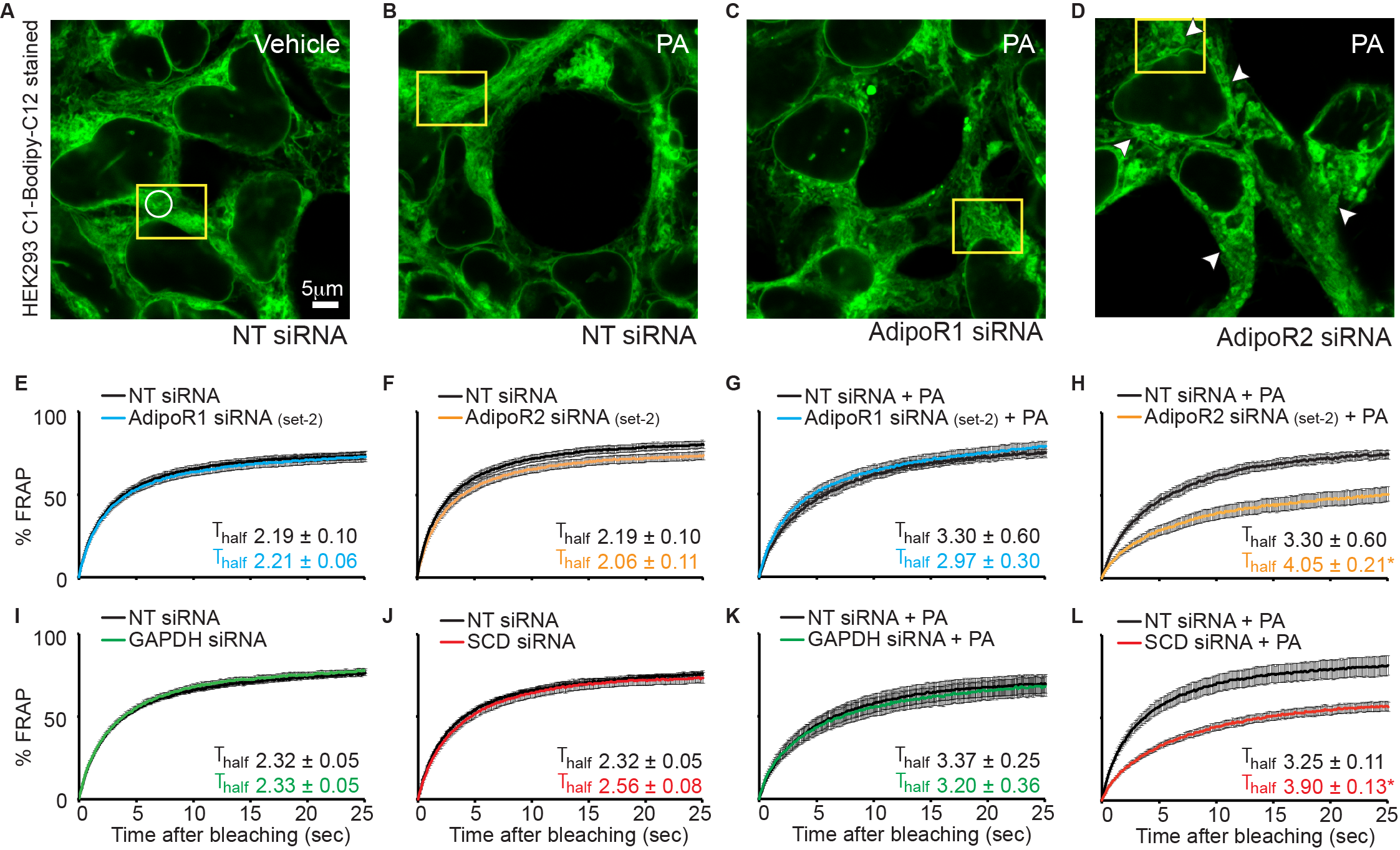

Supplement: S6 Fig — (A-D) The morphology of HEK293 cells is altered by PA when AdipoR2 is knocked down. Note the presence of numerous circular structures in the BODIPY-labeled cells treated with AdipoR2 siRNA. Nuclei are indicated by the letter "N", and the circle in (A) indicates the size of the area that would be bleached in a FRAP experiment. Yellow rectangles indicate the areas enlarged in Fig 6. (E-L) FRAP analysis in HEK293 cells comparing non-target siRNA with siRNA against various genes with or without PA. (TIF) [file pgen.1007004.s006.tif]
